# ADHD symptoms in healthy adults are associated with stressful life events and negative memory bias

**DOI:** 10.1007/s12402-017-0241-x

**Published:** 2017-10-28

**Authors:** Janna N. Vrijsen, Indira Tendolkar, Marten Onnink, Martine Hoogman, Aart H. Schene, Guillén Fernández, Iris van Oostrom, Barbara Franke

**Affiliations:** 1 Department of Psychiatry, Radboud University Medical Center, Donders Institute for Brain, Cognition and Behaviour, PO Box 9101, 6500 HB Nijmegen, The Netherlands; 2 Pro Persona Mental Health Care, Depression Expertise Center, Nijmegen, The Netherlands; 3 Department of Human Genetics, Radboud University Medical Center, Nijmegen, The Netherlands; 4 Department of Cognitive Neurosciences, Radboud University Medical Center, Nijmegen, The Netherlands

**Keywords:** ADHD, Childhood Trauma, Stressful events, Memory bias, Persistence

## Abstract

Stressful life events, especially Childhood Trauma, predict ADHD symptoms. Childhood Trauma and negatively biased memory are risk factors for affective disorders. The association of life events and bias with ADHD symptoms may inform about the etiology of ADHD. Memory bias was tested using a computer task in *N* = 675 healthy adults. Life events and ADHD symptoms were assessed using questionnaires. The mediation of the association between life events and ADHD symptoms by memory bias was examined. We explored the roles of different types of life events and of ADHD symptom clusters. Life events and memory bias were associated with overall ADHD symptoms as well as inattention and hyperactivity/impulsivity symptom clusters. Memory bias mediated the association of Lifetime Life Events, specifically Childhood Trauma, with ADHD symptoms. Negatively biased memory may be a cognitive marker of the effects of Childhood Trauma on the development and/or persistence of ADHD symptoms.

## Introduction

Attention-deficit/hyperactivity disorder (ADHD) is prevalent in children (Polanczyk and Rohde 2007) and persists into adulthood in up to 60% of these young patients, resulting in adult prevalence rates of 2.5–5% (Kessler et al. 2005; Simon et al. 2009). ADHD is characterized by sustained and debilitating symptoms of inattention and/or hyperactivity/impulsivity (Faraone et al. 2015). The diagnostic and statistical manual of mental disorders (DSM; American Psychiatric Association 2013) classification ADHD is considered to represent the extreme of a continuum of traits in the population in children and adults (Chen et al. 2008; Franke et al. 2012; Hoogman et al. 2012; Lubke et al. 2009). ADHD frequently occurs co-morbid with emotional problems, i.e. anxiety disorders and major depression (Herrmann et al. 2010; Uekermann et al. 2010), and this co-occurrence is higher for the inattentive than for the hyperactive/impulsive presentation (Friedrichs et al. 2012).

The aetiology of ADHD is very heterogeneous and there are several developmental pathways leading to the same outcome. A growing body of research links ADHD onset and severity to psychosocial and neurocognitive factors, such as the experience of stressful life events and memory processes. The exposure to stressful life events, and—more specifically—Childhood Trauma, has been shown to predict ADHD onset as well as persistence of the disorder into adulthood (Biederman et al. 1995; Friedrichs et al. 2012; Sugaya et al. 2012), as well as the onset of other psychiatric disorders e.g. depression and anxiety disorders (Hovens et al. 2010; Kessler et al. 1997). In total, some 20–50% of children with a history of Childhood Trauma have clinical levels of ADHD (Glod and Teicher 1996; McLeer et al. 1994). There also is some evidence that ADHD symptom expression may differ between children with and without Childhood Trauma: traumatized children have been found to be less hyperactive (Glod and Teicher 1996). Besides Childhood Trauma, recent stressful events, such as conflicts at work, divorce, and monetary problems, are also associated with levels of ADHD severity (Able et al. 2007; Biederman and Faraone 2006; Sobanski et al. 2007).

Childhood Trauma may result in altered cognitive processing (Perry 2008). Specifically, the cognitive model by Beck (2008) and Beck and Haigh (2014) assumes that the experience of traumatic events during childhood can result in dysfunctional basic assumptions about the self and the world. Stressful events in turn trigger these assumptions. Information is processed in accordance with these assumptions, setting the stage for cognitive biases and increasing the risk for the development of psychiatric problems such as depression. One frequently studied cognitive bias is negative *memory* bias, the preferential and more frequent recall of negative compared to positive material (Mathews and MacLeod 2005). This type of bias is most pronounced for self-relevant material (Steinberg et al. 2003) and is a stable risk factor for subclinical and clinical levels of emotional problems (De Raedt and Koster 2010; Gotlib and Joormann 2010).

Recent evidence from a large naturalistic psychiatric cohort suggests that negative memory bias may be a cognitive marker for a broad range of mental disorders, including ADHD (Vrijsen et al. 2017). Importantly, the association of Childhood Trauma, negative memory bias, and psychiatric problems remained when excluding depressed patients from the analyses, for which the link with bias had earlier been shown. This supports the current proposition that psychiatric disorders share underlying behavioral and neurobiological dimensions (Insel et al. 2010; Insel 2014), one of which is negative memory bias. Regardless of the preliminary evidence for stressful life events in concordance with negative memory bias as global risk factors for psychopathology, biased memory processing of emotional information has only sparsely been directly addressed in ADHD research. A first study reported an association of both inattention and hyperactivity/impulsivity symptoms with memory bias for angry faces compared to happy faces (d’Acremont and Van der Linden 2007). Preliminary evidence for memory bias in ADHD also came from a small study by Krauel et al. (2009), who found that adolescents with ADHD and comorbid externalizing problems showed less positive memory bias compared to healthy controls and ADHD-only participants. Examining memory bias in relation to ADHD symptoms may inform about the aetiology of ADHD. Moreover, cognitive markers—i.e. memory biases—offer a relatively easy target for (computerized) treatment (Hertel et al. 2017; Koster and Bernstein 2015; Mathews and MacLeod 2002; Vrijsen et al. 2016). Hence, examining the role of memory bias in ADHD may also aid in the development of treatment tools.

To explore the relevance of the cognitive model (Beck 2008; Beck and Haigh 2014) for ADHD and improve our knowledge about the relation between stressful life events, memory bias, and ADHD, we here performed association studies in a large sample of self-reported healthy adults, which provided information over a large range of ADHD symptom severity (Hoogman et al. 2012). In line with the cognitive model (Beck 2008; Beck and Haigh 2014) and following association studies of the different individual components, we assessed the possible mediation of the association between life events and self-reported inattention and hyperactivity/impulsivity symptoms by memory bias. Given the broad array of stressful life events—ranging from childhood sexual abuse to financial problems—found associated with adult ADHD (symptom severity) in previous studies, we first examined the association of lifetime stressful events (lifetime life stress) with ADHD symptoms as well as mediation of this association by memory bias. Our second aim was to explore the specificity of type of life events on ADHD symptoms. Hence, Childhood Trauma (early life stress) and recent stressful events (recent life stress) were both association with ADHD symptom severity. Because both the presence as well as the diversity of stressful events has been related to psychopathology (Hovens et al. 2010; Vrijsen et al. 2017), we used variables representing the number of events. We also explored the association between life events and inattention and hyperactivity/impulsivity symptom clusters separately. The results will provide a first indication of the relevance of Childhood Trauma and memory bias for ADHD symptoms and may instigate further research.

## Methods and materials

### Participants

The study was performed using the Cognomics Initiative Resource, the Brain Imaging Genetics (BIG) sample (http://www.cognomics.nl), which consists of self-reported healthy mainly young adults. Subsamples of the current sample have previously been described elsewhere (e.g. Hoogman et al. 2012; van Oostrom et al. 2012; Vogel et al. 2014; Vrijsen et al. 2015a). Participants were screened using a self-report questionnaire for the following exclusion criteria: history of somatic disease potentially affecting the brain, current or past psychiatric or neurological disorder, use of medication (except hormonal contraceptives) or illicit drug(s) during the past 6 months, history of substance abuse, current or past alcohol dependence, current pregnancy or lactation, and menopause. All participants were fluent in Dutch. A total of 785 participants completed an ADHD symptom questionnaire (see below; Kooij et al. 2005). Of those participants, memory bias data was available for 675 individuals. The BIG study was approved by the regional medical ethics committee. All participants gave written informed consent and were financially compensated for participation.

### ADHD symptoms

The ADHD DSM-IV-TR Rating Scale for use in adults was used to assess current ADHD symptoms (Kooij et al. 2005). This instrument has shown internal and external validity in a large population-based adult sample (Kooij et al. 2005). Symptoms in the last 6 months were reported on a 4-point scale. A symptom was considered to be present if participants answered ‘often’ or ‘very often’. The scores on the 23 items were recalculated to the original 18 DSM-IV-TR ADHD criteria, of which nine criteria are related to the inattention (IA) symptom domain and nine to the hyperactivity/impulsivity (HI) symptom domain. The variables ‘Total ADHD’ symptoms (possible range 0–18),’ IA-symptoms’ (range 0–9), and ‘HI-symptoms’ (range 0–9) were derived from the data (for more details see Hoogman et al. 2012). The Total ADHD variable, as well as the IA-symptoms and the HI-symptoms subscales had acceptable reliability in the current sample, *α* = .78, *α* = .72, and *α* = .64, respectively.

### Positive and negative affective state

The Positive and Negative Affect Schedule (PANAS; Watson et al. 1988) was used to assess affective state. The PANAS including its subscales have been shown to be reliable and valid for measuring positive and negative affect in a large non-clinical sample (Crawford and Henry 2004). This instrument comprises two mood scales, one for the assessment of positive affect and one for negative affect. Ten descriptors are used for each scale to define their meanings, resulting in a 20-item questionnaire using a 5-point scale that ranges from ‘very slightly or not at all’ to ‘extremely’. Because of the relevance for memory bias and risk for psychopathology (e.g. Bower 1981), we selected the negative affect subscale (possible range 10–50) for this study. This subscale has been found to correlate highly with depressive symptom levels (Tarlow and Haaga 1996). This subscale was found to be highly reliable in the current sample (10 items; *α* = .88).

### Stressful life events

Stressful life events were assessed with an adapted version of the List of Threatening Evens Questionnaire (Brugha and Cragg 1990). The original instrument has good test–retest reliability, high agreement between participant and informant ratings, as well as good agreement with interview-based ratings specificity (Brugha and Cragg 1990). The adapted instrument has not been validated, but has previously been used in studies on biased processing including patient studies (e.g. van Oostrom et al. 2012; Vogel et al. 2014; Vrijsen et al. 2014a, b). Participants were asked to indicate, whether they had experienced a set of 21 life events before the age of 16 years, after the age of 16, and/or within the last year. In line with the study by Vogel et al. (2014), a ‘Lifetime Life Events’ variable was calculated, indicating the total number of experienced life events. Based on previous studies (van Oostrom et al. 2012; Vrijsen et al. 2014a, b, 2015b), a ‘Childhood Trauma’ variable and a ‘Recent Stress’ variable were also calculated. ‘Childhood Trauma’ indicated the number of different traumatic events (aggression, sexual, and/or physical abuse) the participant experienced within or outside the family before the age of 16 years. ‘Recent Stress’ reflected the number of different stressful events (health problems, health problems of a close one, death of a family member, problems within the romantic relationship, divorce, a conflict at work, monetary problems, or legal issues) the participant experienced within the last year.

### Memory bias

Memory bias was assessed using a web-based version of the self-referent encoding/evaluation task (SRET; Hammen and Zupan 1984). The SRET has been used in previous publications from BIG (Gerritsen et al. 2012; van Oostrom et al. 2012; Vogel et al. 2014; Vrijsen et al. 2015). The task had explicit instructions. During encoding, 12 negative and 12 positive trait adjectives were presented one by one on a screen in fixed random order. Participants were instructed to indicate for each word, whether it was self-referent or not by pressing one of two buttons. Following a 2.5 min distraction task (mental arithmetic), participants were asked to type in as many of the words they could remember from the encoding phase within 3 min. Responses to the first and last two words of the word list were excluded from analysis in order to avoid primacy and recency effects. Incorrect responses were checked manually, and spelling errors as well as plurals if the original word was singular (and vice versa) were permitted.

In line with previous the studies using this task, two outcome variables were calculated: proportion of self-referent negative recall (negative memory bias) and proportion of self-referent positive recall (positive memory bias). The positive and negative memory bias variables were calculated by dividing the number of adjectives endorsed as self-referent and recalled in a given valence category by the total number of self-endorsed adjectives.

### Statistical analyses

Age and gender were entered as covariates in the mediational model analyses based on the associations of these variables with the independent variables: Lifetime Life Events and memory bias. The PANAS negative affect scale total score was added as covariate in all analyses to correct for variation due to differences in negative ‘depressotypic’ state. Prior to analyses, log transformation was applied in case of non-normally distributed scores. After transformation, the values for asymmetry all lay between − 2 and + 2, and were hence acceptable in order to prove normal univariate distribution (George and Mallery 2010). One-tailed bivariate correlations were calculated between the life events variables, ADHD symptoms, and positive and negative memory bias. For these correlations, Bonferroni multiple testing correction was based on the number (6) of comparisons among life events, positive bias, negative bias, and ADHD symptom variables, resulting in a significance level of *p* < .0083. Based on the correlations (see Table 2) and to assess the overall relevance of life events for ADHD symptom levels, the mediation of the association of Lifetime Life Events with total ADHD symptoms by memory bias was tested. Associations as well as mediation were also tested separately for the IA and HI symptom clusters to get an indication of potential specificities of findings. Subsequent model were constructed testing the mediation of the association of Childhood Trauma with total ADHD symptoms as well as the symptom clusters by memory bias. Mediation was tested using the PROCESS macro for SPSS (Hayes 2013). A bootstrapping method was used to assess the indirect effect based on 1000 bootstrapped samples using bias-corrected and accelerated 95% confidence intervals (BCa CI).

## Results

### Sample descriptives and correlational analyses

Complete data were available for 675 participants. Descriptives of the sample are shown in Table 1. Age was significantly correlated to the number of Lifetime Life Events, *r*(673) = .22, *p* < .001, but not to the memory bias variables, all *p* > .07. When comparing men and women on the number of Lifetime Life Events and memory bias, we saw gender differences on both the positive and negative memory bias scores, *t*(673) = 3.33, *p* = .001 and *t*(673) = 2.02, *p* = .044, respectively.^1^

**Table 1:** Sample descriptives including means (standard deviations) or percentages and range and absolute numbers for the variables: age, sex, negative affect, life events, and ADHD symptoms (*N* = 675)

Variable	Mean (SD) and range and absolute numbers; or % of total
Age in years	22.7 (3.8), range 18–39
Sex (% female)	62%
PANAS negative affect score	13.5 (4.8), range 10–47
Experienced Lifetime Life Events	99%
Number of different Lifetime Life Events	5.1 (3.3), range 0–23
Experienced Childhood Trauma	21%
Number of different Childhood Trauma	0.3 (0.6), range 0–3
0	536
1	104
2	28
3	7
Experienced recent stressful events	30%
Number of different recent stressful events	0.4 (0.1), range 0-4
0	474
1	137
2	53
3	9
4	2
Total ADHD symptoms	2.9 (2.9), range 0–16
ADHD inattention symptoms	1.2 (1.7), range 0–9
ADHD hyperactivity/impulsivity symptoms	1.7 (1.7), range 0–8

The correlational structure of the independent and dependent variable data is given in Table 2. As apparent, negative memory bias, but not positive memory bias, was significantly positively correlated with total ADHD symptom level (*p* < .001); both inattention and hyperactivity/impulsivity symptoms contributed to this correlation. A significant positive correlation was also seen between the Lifetime Life Events and Recent Stress variables, and total ADHD score (both *p* < .001). Both inattention and hyperactivity/impulsivity symptoms contributed to the correlation with Lifetime Life Events. Hyperactivity/impulsivity symptoms but not inattention symptoms were significantly correlated with Recent Stress (*p* = .001 and *p* = .210, respectively). Furthermore, positive correlations were found for negative memory bias and Lifetime Life Events as well as Childhood Trauma (*p* < .005 for both), but not Recent Stress. None of the three life events variables correlated with positive memory bias. Therefore, mediation analyses were only run for negative memory bias. Also, because of the absence of correlations between Recent Stress and negative memory bias variable, mediation of the association of Recent Stress and ADHD symptoms by negative memory bias was not examined.

**Table 2:** Bivariate correlations (including *p*-values) between stressful life events variables, positive and negative memory bias, and ADHD symptom level (including subscales) variables (*N* = 675)

	1.	*1.1*	*1.2*	2.	3.	4.	*4.1*	*4.2*	
1. LT Life Events	–								
1.1 CH Trauma	.46, *p* < .001*	–							
1.2 RC Stress	.47, *p* < .001*	.13, *p* < .001*	–						
2. Positive bias	− .03, *p* = .254	− .06, *p* = .055	.02, *p* = .322	–					
3. Negative bias	.16, *p* < .001*	.12, *p* = .001*	.05, *p* = .095	− .09, *p* = .007*	–				
4. Total ADHD symp.	.15, *p* < .001*	.11, *p* = .002*	.11, *p* = .002*	− .04, *p* = .165	.23, *p* < .001*	–			
4.1 IA symp.	.12, *p* = .001*	.08, *p* = .019	.03, *p* = .210	− .06, *p* = .073	.19, *p* < .001*	.80, *p* < .001*	–		
4.2 HI symp.	.14, *p* = .001*	.11, *p* = .003*	.02, *p* = .001*	.01, *p* = .367	.20, *p* < .001*	.87, *p* < .001*	.44, *p* < .001*	–	

*IA* inattention symptom score, *HI* hyperactivity/impulsivity symptom score, *LT* Life time, *CH* childhood, *RC* recent

* Significant at the *p* < .0083 level (*p*-level threshold after correction for multiple testing)

### Life events and ADHD symptoms—mediation by negative memory bias

**Fig. 1:**
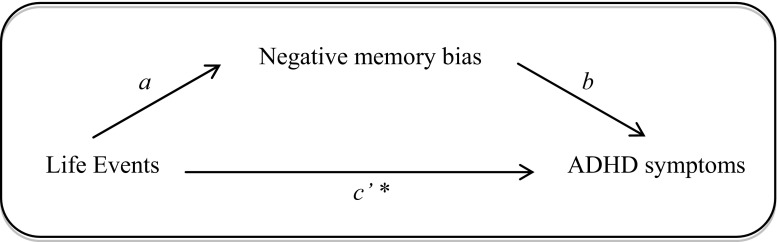
Model for the relationship between life events and ADHD symptoms as mediated by negative memory bias. *The regression coefficient between life events and ADHD symptoms when negative memory bias is included in the model. *a* = regression coefficient of the association between Life Events and Negative memory bias, b = coefficient of the association between Negative memory bias and ADHD symptoms, c′ = estimate of the direct effect of Life Events on ADHD symptoms

In the following, negative memory bias was examined as a mediator of the association between life events and ADHD symptoms according to the model presented in Fig. 1. Indeed, the relationship between Lifetime Life Events and total ADHD symptoms was found to be mediated by negative memory bias: the regression coefficients between Lifetime Life Events and negative memory bias as well as between negative memory bias and total ADHD symptoms were statistically significant. In addition, the association between Lifetime Life Events and total ADHD symptoms stayed significant, when allowing for mediation by negative memory bias, with *p* < .001 in the total effect model. The indirect effect ‘ab’ was small (0.005), but statistically significant as the bootstrapped unstandardized indirect effect 95% confidence interval (BCa CI) ranged from .002 to .01 (i.e. not including zero). The ratio of the indirect effect (referred to as P_M_) to the direct effect was .18. The P_M_ value provides an effect size measure, and in this case it indicates that 18% of the effect of life events on ADHD symptoms operates indirectly through negative memory bias.

### Model specification for IA and HI ADHD symptom clusters

In separating the ADHD total scores into scores for the two different symptom domains, we found both the associations of Lifetime Life Events with inattention (IA) symptom score as well as with hyperactivity/impulsivity (HI) symptom score to be mediated by negative memory bias (see Table 3: Model 2 and 3, respectively with BCa CI [.001, − .01] for both models). The strength of effect appeared similar for the two symptom clusters (see coefficients in Table 3). P_M_ was .21 for Model 2 and .18 for Model 3.

**Table 3:** Regression coefficients, standard errors, and model summary information for the mediational models testing the association between the life events variables (lifetime and Childhood Trauma), negative memory bias, and ADHD symptom level (including the IA and HI subscales) controlled for the effect of age, gender, and negative affect (*N* = 675). The tested model is depicted in Fig. 1

Antecedent	Consequent							
	*M* (Negative memory bias)				Y (ADHD symptoms)			
		Coeff.	SE	*p*		Coeff.	SE	*p*
Model 1: Lifetime Life Events and total ADHD symptoms								
* X* (Lifetime Life Events)	*a*	0.004	0.001	< .001	*c′*	0.027	0.008	.002
* M* (Neg. memory bias)		–	–	–	*b*	1.254	0.313	< .001
Constant	*i*_*M*_	− 0.024	0.023	0.289	*i*_*Y*_	0.586	0.128	.001
		*R* ^*2*^ = 0.076 *F*(4670) = 13.699, *p* < .001				*R* ^*2*^ = 0.145 *F*(5669) = 22.673, *p* < .001		
Model 2: Lifetime Life Events and IA ADHD symptoms								
* X* (Lifetime Life Events)	*a*	0.004	0.001	< .001	*c′*	0.018	0.007	.016
* M* (Neg. memory bias)		–	–	–	*b*	.986	0.273	< .001
Constant	*i*_*M*_	− 0.024	0.023	.289	*i*_*Y*_	0.276	0.159	.084
		*R* ^*2*^ = 0.076 *F*(4670) = 13.699*, p* < .001				*R* ^*2*^ = 0.119 *F*(5699) = 18.023*, p* < .001		
Model 3: Lifetime Life Events and HI ADHD symptoms								
* X* (Lifetime Life Events)	*a*	0.004	0.001	< .001	*c′*	0.019	0.007	.008
* M* (Neg. memory bias)		–	–	–	*b*	.906	0.267	< .001
Constant	*i*_*M*_	− 0.024	0.023	.289	*i*_*Y*_	0.301	0.156	.054
		*R* ^*2*^ = 0.076 *F*(4670) = 13.699*, p* < .001				*R* ^*2*^ = 0.118 *F*(5699) = 18.898*, p* < .001		
Model 4: Childhood Trauma and total ADHD symptoms								
* X* (Childhood Trauma)	*a*	0.030	0.010	.002	*c′*	0.148	0.078	.059
* M* (Neg. memory bias)		–	–	–	*b*	1.323	0.313	< .001
Constant	*i*_*M*_	− 0.028	0.023	.221	*i*_*Y*_	0.567	0.184	.002
		*R* ^*2*^ = 0.070 *F*(4670) = 12.607, *p* < .001				*R* ^*2*^ = 0.317 *F*(5669) = 21.157*, p* < .001		
Model 5: Childhood Trauma and IA ADHD symptoms								
* X* (Childhood Trauma)	*a*	0.030	0.010	.002	*c′*	0.063	0.068	.353
* M* (Neg. memory bias)		–	–	–	*b*	1.049	0.273	< .001
Constant	*i*_*M*_	− 0.028	0.023	.221	*i*_*Y*_	0.267	0.160	.096
		*R* ^*2*^ = 0.070 *F*(4670) = 12.607, *p* < .001				*R* ^*2*^ = 0.112 *F*(5669) = 16.914, *p* < .001		
Model 6: Childhood Trauma and HI ADHD symptoms								
* X* (Childhood Trauma)	*a*	0.030	0.010	.002	*c′*	0.135	0.067	.043
* M* (Neg. memory bias)		–	–	–	*b*	0.942	0.267	< .001
Constant	*i*_*M*_	− 0.028	0.023	.221	*i*_*Y*_	0.284	0.156	0.070
		*R* ^*2*^ = 0.070 *F*(4670) = 12.607, *p* < .001				*R* ^*2*^ = 0.114 *F*(5669) = 17.239, *p* < .001		

All of the indirect effects of *X* on *Y* were statistically significant, which means evidence for mediation was found in all models. This was revealed by the bootstrapped Confidence Intervals not including zero in all models

*IA* inattention symptom score, *HI* hyperactivity/impulsivity symptom score, *X* predictor life events variable, *Y* outcome variable, ADHD symptom variable, *M* mediator, negative memory bias; *a* = regression coefficient of the association between life events and negative memory bias, *b* coefficient of the association between negative memory bias and ADHD symptoms, *c’* estimate of the direct effect of life events on ADHD symptoms, *i* constant coefficient

### Childhood Trauma and ADHD symptoms

When examining the associations with Childhood Trauma only (Model 4), we found that the direct effect of Childhood Trauma on negative memory bias was significant, but the direct effect of Childhood Trauma on total ADHD symptoms fell short of reaching statistical significance, with *p* = .059.^2^ Nevertheless, negative memory bias still seemed to mediate some aspects of Childhood Trauma on total ADHD symptoms with BCa CI [.01, − .08], as the indirect effect in the total effect model was significant, and 27% of the effect of Childhood Trauma on ADHD symptoms seems to operate indirectly through negative memory bias.

For the separate ADHD symptom domains, we observed an interesting pattern. As apparent from Table 3, Models 5 and 6 (BCa CIs [.01, − .08] and [.01, − .06], respectively), effects of Childhood Trauma on inattention severity operate largely through memory bias (P_M_ = .49): early trauma strongly affected negative memory bias, but no significant direct effects on inattention symptoms were observed. For hyperactivity/impulsivity the pattern was different, with significant direct and indirect effects, and mediating memory bias explaining only approximately 21% of the effect.

## Discussion

The current study substantiates earlier suggestive findings showing associations between life events and population ADHD symptoms, and shows that such associations might be particularly driven by childhood traumatic events. The associations were robust, when controlling for negative ‘depressotypic’ affect, and were observed for both inattention and hyperactivity/impulsivity. We provide new evidence for the mediation by negative memory bias of associations between life events and population ADHD symptom scores. This first exploration of the relevance of memory bias as marker for ADHD symptoms will hopefully stimulate further (sub)clinical research into cognitive biases in ADHD.

Associations between Lifetime Life Events and ADHD symptoms seemed to be driven mainly by childhood traumatic events. Indeed, Childhood Trauma has a strong effect on the developing child, at the neural, cognitive, and emotional level (e.g. Biederman et al. 1995; Stein et al. 1997; Sugaya et al. 2012) as well as being a known risk factor for the development of ADHD symptoms (e.g. Stevens et al. 2008). In addition and conversely, children showing high ADHD symptom levels may be particularly prone to experiencing Childhood Trauma such as aggression or abuse, because they tend to act out more and/or because of their family members might show problems with impulse control themselves (ADHD has a strong genetic basis; Franke et al. 2012; Harold et al. 2013). Although we had also hypothesized a possible association between Recent Stress and ADHD symptoms, given earlier reports of more Recent Stress on ADHD severity in a clinical sample (van der Meer et al. 2014), the observed nonsignificant correlations did not provide evidence for such a link in our population sample. A potential explanation might be that the correction for negative affect scores removed much of the variance related to Recent Stress. We also employed a rather conservative significance level for the correlations, and it may be important to note that the direct association between Recent Stress and ADHD symptoms (total and hyperactivity/impulsivity scores) did reach an uncorrected level of significance of *p* < .05. Although our results cannot address the direction of the association, they may indicate that while recent stressors can still exacerbate current symptom severity somewhat, Childhood Trauma—possibly through memory processes—may be a risk factor for both the development and persistence of ADHD symptoms into adulthood. The latter is important, since we still lack a good understanding of the factors that influence persistence of ADHD symptoms into adulthood. Persistence has for example been associated with childhood ADHD severity and whether or not patients received treatment during childhood (Kessler et al. 2005), as well as neural and cognitive processes (e.g. Alderson et al. 2013; Onnink et al. 2014). Understanding (and in the future possibly predicting) outcome of ADHD in adulthood is crucial to inform treatment options, clinical planning, and lifestyle choices of individuals at risk.

Though mediation of negative memory bias was seen for the association between Childhood Trauma and total ADHD symptoms, it was surprisingly more specific for hyperactivity/impulsivity levels in this population cohort. Childhood Trauma affects brain areas vital for cognitive control and memory functioning (Bremner 2002; Bremner and Narayan 1998; Bremner et al. 2003; McGaugh 2004; Stein et al. 1997). This may result in less effective suppression of unwanted negative material in memory, resulting in memory bias (Nolen-Hoeksema et al. 2008). Besides negative memories, the neural effects might—directly or through memory bias—be associated with less effective suppression of hyperactive and impulsive tendencies. To find out whether this interpretation is relevant for the clinical extreme of hyperactivity/impulsivity, substantiation by studies in clinical samples is required.

Important to note is that, because of the cross-sectional design of our study, we could not study causation and currently cannot know the direction of the effects observed. Our model was built under the assumption that memory bias can be a marker for ADHD, but studies in e.g. major depression show that levels of memory bias can also be influenced by Recent Stress (Gotlib and Joormann 2010). Memory bias is thus partly state-dependent, but through controlling for negative affect we seem to tap into trait-like characteristics of negative memory bias. As we also did not find effects of recent life events, the model constructed and shown in Fig. 1 is the most likely model explaining the relationship between life events, memory bias, and ADHD symptoms. The development and persistence of ADHD symptoms in the population may have an ‘emotional’ component, akin to anxiety and depression. In fact, ADHD shares some symptoms with these disorders, e.g. avoidance and cognitive impairment. Perhaps negative cognitive schemata (Beck 2008), based on childhood experiences, thus play a role in the development of neurodevelopmental disorders as well (see also recent evidence by Vrijsen et al. 2017). We present a first evaluation of the relevance of life stress and memory bias for ADHD symptoms in a healthy sample. Important to note is that the effect sizes of the correlations are small, which is not surprising given the limited variance in ADHD symptoms in this healthy sample. Substantiation in a subclinical an eventually clinical sample are future steps. If further substantiated, addressing schemata in cognitive behavioral therapy or computerized cognitive trainings (i.e. Cognitive Bias Modification; Koster and Bernstein 2015) for ADHD might be beneficial.

Our study should be viewed in the context of some strengths and limitations. Strength of the study is the large sample of participants. This is especially important, when examining novel markers for a given disorder. We used a population sample not containing diagnosed patients with ADHD for our study, which afforded us the possibility to study the effect of symptoms severity along the entire continuum observed in the population. We view this as a strength, but it also requires confirmation of findings in a clinical sample, before any we can start making inferences for disease and treatment. We studied two classes of stressful life events (i.e. Recent Stress and Childhood Trauma), adding to the understanding of the role of Childhood Trauma in ADHD. However, the variance of recent stressful events and Childhood Trauma is limited in a healthy sample. Web-based data collection was used, which limits the controllability of data collection. However, testing participants in their natural setting eliminates experimenter effects (Harris and Rosenthal 1985), in turn increasing the ecological validity of the results. The measure of life events provides a limitation, as trauma severity and subjective stress associated to the traumas were not measured. Another possible limitation of the measurement method for the life events is that negative bias might have affected participants’ recall. Because factual events during pre-determined periods of life were assessed, and because adults’ recall of childhood events has been shown to be fairly accurate (Brewin et al. 1993), it seems unlikely that this would have significant effects on our study beyond potentially adding some random noise, limiting power.

In conclusion, biased processing of emotional information may be a marker for adult ADHD symptom severity in addition to being a risk factor for depression onset, maintenance, and recurrence (De Raedt and Koster 2010). Memory bias association with ADHD symptoms was independent of current depressive mood state, and more negative memory bias was linked to increased levels of both inattention and hyperactivity/impulsivity. The current results make the study of cognitive biases in neurodevelopmental disorders an interesting new research direction.

## Footnotes

1 The PANAS negative affect scale was significantly positively correlated with the number of Lifetime Life Events (*r* = .08), Recent Events (*r* = .13), total ADHD symptom levels (*r* = .34) as well as both IA (*r* = .27) and HI subscales (*r* = .31), and the negative memory bias score (*r* = .22), all *p* < .05. Negative affect was not significantly correlated with Childhood Trauma variable, *r*(673) = .07, *p* = .079.

2 The pattern of results is similar when using the dichotomized Childhood Trauma variable (yes vs. no trauma) in the mediational models. All of the indirect effects of Childhood Trauma on ADHD symptoms (total, IA, and HI subscales) were statistically significant, which means evidence for mediation was found in all models. This was revealed by the bootstrapped confidence intervals not including zero in all models. The only difference with the models including the continuous Childhood Trauma variable was that the direct effect on HI symptoms did not reach statistical significance using the dichotomous Childhood Trauma variable (*p* = .068).
